# When Is a Reaction Network a Metabolism? Criteria for Simple Metabolisms That Support Growth and Division of Protocells

**DOI:** 10.3390/life11090966

**Published:** 2021-09-14

**Authors:** Paul G. Higgs

**Affiliations:** Department of Physics and Astronomy, Origins Institute, McMaster University, Hamilton, ON L8S 4M1, Canada; higgsp@mcmaster.ca

**Keywords:** metabolism, autocatalytic set, origin of life, osmotic pressure, cell division, lipid membrane, bistable reaction system, template-directed RNA synthesis

## Abstract

With the aim of better understanding the nature of metabolism in the first cells and the relationship between the origin of life and the origin of metabolism, we propose three criteria that a chemical reaction system must satisfy in order to constitute a metabolism that would be capable of sustaining growth and division of a protocell. (1) Biomolecules produced by the reaction system must be maintained at high concentration inside the cell while they remain at low or zero concentration outside. (2) The total solute concentration inside the cell must be higher than outside, so there is a positive osmotic pressure that drives cell growth. (3) The metabolic rate (i.e., the rate of mass throughput) must be higher inside the cell than outside. We give examples of small-molecule reaction systems that satisfy these criteria, and others which do not, firstly considering fixed-volume compartments, and secondly, lipid vesicles that can grow and divide. If the criteria are satisfied, and if a supply of lipid is available outside the cell, then continued growth of membrane surface area occurs alongside the increase in volume of the cell. If the metabolism synthesizes more lipid inside the cell, then the membrane surface area can increase proportionately faster than the cell volume, in which case cell division is possible. The three criteria can be satisfied if the reaction system is bistable, because different concentrations can exist inside and out while the rate constants of all the reactions are the same. If the reaction system is monostable, the criteria can only be satisfied if there is a reason why the rate constants are different inside and out (for example, the decay rates of biomolecules are faster outside, or the formation rates of biomolecules are slower outside). If this difference between inside and outside does not exist, a monostable reaction system cannot sustain cell growth and division. We show that a reaction system for template-directed RNA polymerization can satisfy the requirements for a metabolism, even if the small-molecule reactions that make the single nucleotides do not.

## 1. Introduction

One of the long-standing questions in the area of the origin of life is whether the origin of replication of information-carrying polymers occurred before or after the origin of metabolism [1,2,3]. On the replication-first side, there is considerable experimental progress with non-enzymatic RNA replication [4,5], with polymerase ribozymes [6,7], and with the encapsulation of RNAs inside artificial protocells [8,9,10,11,12,13]. On the metabolism-first side, there is very little experimental evidence that small-molecule reactions can support cell maintenance, growth and division in the absence of genetic polymers. Although computational models of small-molecule assemblies can show compositional inheritance without having genetic sequences [14], there are persistent doubts as to whether such systems could evolve [15]. Since the capacity for Darwinian evolution is usually considered to be a defining feature of life [16], this would preclude any small-molecule autocatalytic network from being defined as life. However, whatever scenario we favour for the origin of life, we must acknowledge that metabolism is a key part of the puzzle.

There is no generally agreed definition of metabolism that is appropriate for research work in the origin of life. For the present purposes, we will simply define a metabolism as a chemical reaction system that can sustain growth and division of a cell. In our view, progress in understanding the origin of metabolism has been hampered by lack of a clear understanding of what properties a reaction system must have in order to constitute a metabolism. The aim of this paper is therefore to define criteria that a reaction system must satisfy in order to be able to support growth and division of a protocell, and to give examples illustrating the difference between systems that do and do not satisfy these criteria.

Theoretical models of metabolism go back to chemotons [17,18], which emphasize that the metabolic system is inside a membrane whose growth must also be sustained by the metabolism. Another important conceptual step was to show that an autocatalytic set of molecules is likely to exist in models of random reaction networks [19]. This led to the model of reflexively autocatalytic and food-generated (RAF) sets [20,21,22,23], which has been widely used recently to describe metabolic networks. An RAF set is a set of molecules and reactions such that every molecule is either a food molecule present in the environment or a biomolecule formed by the reactions, and such that every reaction is catalyzed by a molecule in the set. The expectation is that this set of molecules can maintain itself, given a continued supply of the food molecules. However, in our opinion there are several problems with the RAF model that make it incomplete as a theory. The requirement that every reaction be catalyzed by another molecule seems too strong when we are dealing with simple metabolisms in the earliest forms of life. In modern organisms, almost every reaction of small-molecule metabolites is catalyzed by enzymes. However, prior to the existence of RNA and proteins, there were no enzymes, so we need a theory that deals with the reactions of small molecules without insisting that the reactions be catalyzed. A further issue with the RAF model is that most of the work with this theory deals only with the structure of the reaction network, and not with rates and concentrations. The criteria for metabolisms that we use in this paper cannot be assessed without knowledge of rates and concentrations.

Another type of model that has been developed recently treats metabolic networks as cyclic processes [24,25,26,27,28,29,30]. In this framework, catalysts are treated on an equal footing with other molecules in the system, and all the steps in a catalytic process are specified. For example, suppose the reaction A+B→AB is catalyzed by another molecule C. In the RAF framework, this reaction would be treated as a single reaction that is assumed to occur if C is present, but the mechanism by which C controls the reaction would not be specified. The influences of catalysts on reactions are drawn as dashed arrows in the diagrams that represent RAF sets [20,21,22,23]. The way these influences operate is left unspecified. In contrast, in the cyclic process framework, the mechanism by which the catalyst participates in the process is specified. For example, the formation of AB could occur via two steps that involve C: C+A→CA;CA+B→C+AB. This shows that the catalyst C is regenerated unchanged at the end, and it also shows that the mechanism requires formation of another molecule CA, whose existence would not be known if the reaction were written as a single step.

If C were an enzyme catalyst, this mechanism would resemble the Michaelis–Menten theory of enzyme kinetics. The enzyme–substrate complex in the Michaelis–Menten theory (analogous to CA in this example) is usually considered to be a temporary physical association of the substrate to the enzyme surface. However, if we are dealing with small-molecule reactions, then physical associations seem less likely, and the formation of CA is more likely a chemical reaction involving chemical bond formation. The reaction C+A→CA is a chemical reaction that should be treated on the same footing as the original reaction A+B→AB. If we say that the formation of AB  is catalyzed, it means that the direct formation reaction is slow or does not occur, but the steps involving C are faster. In this framework, it does not make sense to insist that every reaction is catalyzed, otherwise we would need two additional catalysts to catalyze two the steps involving C, and further catalysts to catalyze these catalysts, and so on ad infinitum. Note that there is more than one way in which C could catalyze the first reaction. It could react with B before A: B+C→BC;A+BC→AB+C. Or the process could involve three steps instead of two: C+A→CA;CA+B→CAB;CAB→C+AB. The dynamics of the reactions and the concentrations of the molecules will depend on the mechanism by which C participates in the process.

In this paper, we consider examples of reaction networks inside a cellular compartment that are described in the cyclic process framework. Transport of some types of molecule into and out of the cell is possible by diffusion through a semipermeable membrane, but the membrane may be impermeable to other kinds of molecules. We will divide molecules involved into three types: food molecules, biomolecules and waste molecules. Food molecules are present in the external environment and are imported into to the cell. Biomolecules are formed inside the cell and they remain at low or zero concentration outside the cell. The presence of these biomolecules at a high concentration inside the cell is one factor that distinguishes the living state inside from the non-living state outside. Waste molecules are those produced inside the cell which do not play a constructive role in further reactions in the cell and are exported from the cell.

The state with low biomolecule concentration outside the cell must be stable to perturbations that add small amounts of these molecules to the outside. Although it is possible in principle to begin with zero concentration of biomolecules outside and allow biomolecules to be present only inside, there will always be some possibility of leakage of small amounts of biomolecules from the inside to the outside, either through a small rate of permeability through the membrane or by occasional bursting of a cell. When this occurs, the external concentration of biomolecules must return to its original low or zero concentration. If the non-living state were not stable to small perturbations of this kind, the external environment would shift spontaneously to the living state, and there would no longer be any difference between the inside and outside.

When the reaction system maintains a positive osmotic pressure inside the cell, i.e., the total solute concentration is higher inside than outside, the osmotic pressure can drive growth of the system if the boundary is flexible. For a system enclosed in a lipid membrane, growth of the membrane surface area is possible if there is a supply of lipid available outside the cell or if new lipid is synthesized inside the cell by the reaction system itself.

The reaction system cannot be in thermodynamic equilibrium. Inside the cell, there must be a steady state with continual turnover, consisting of import of food molecules, conversion of food to biomolecules, decay of biomolecules to waste, and export of waste. If the inside were in equilibrium, all reactions would be balanced by equal and opposite reactions, and there would be no net flow of material through the system. In order to drive flow through the system inside the cell, the system outside must also be maintained out of equilibrium, i.e., the food molecules must have a higher concentration and the waste molecules must have a lower concentration that they would do if all reactions were in equilibrium. This is possible if some of the reactions that convert food molecules to biomolecules cannot occur outside the cell, or occur very slowly. If they do not occur at all, then food molecules can remain out of equilibrium at high concentration with no flow of material though the system. If the reactions occur slowly, then there will be a steady-state flow through the external reaction system that must be maintained by an environmental recycling or replenishment process (supply of food and removal of waste). The rate of the turnover due to environmental recycling outside can be compared with rate of the turnover due to metabolism inside the cell. We expect the turnover rate inside the cell to be higher than outside, otherwise the outside is “more alive” than the inside. In what follows, we define the metabolic rate as the rate of mass transfer per unit volume from food molecules to biomolecules. The volume of the external environment will be much larger than that of the cell. The per unit volume consumption rate of the food molecules outside the cell must be small, otherwise a massive supply rate of food molecules would be required that could not be maintained by an environmental recycling process.

We summarize the above considerations by the following three criteria which must be satisfied if the reaction network is to be considered as a metabolism that can support growth and division of a cell.

1. The reaction system should maintain a state of high biomolecule concentration inside the cell and low biomolecule concentration outside, and these states should be stable at the same time. Small perturbations should not cause either the death of the internal living system or the spontaneous springing to life of the external non-living system.
2. The total concentration inside the cell should be higher than outside, so that a positive osmotic pressure is maintained in the cell. This can drive growth of the cell if the boundary is flexible and lipid molecules are available to form new membrane.
3. The metabolic rate of the reaction system can be defined as the rate of mass flow from food molecules to biomolecules. The metabolic rate inside the cell should be higher than outside the cell.

These requirements can be satisfied if the reaction system is bistable. In this case, the reactions have the same rate constants inside and outside the cell, but there are two different steady-state concentrations of the molecules. The requirements can also be satisfied if the reaction system is monostable and if some of the reactions have different rate constants inside and out. For example, the decay rate of biomolecules to waste might be high on the outside and low on the inside, or the formation rates of biomolecules might be high on the inside and low on the outside. In this case, the difference in concentrations between the inside and outside can be maintained, but it is necessary to propose some additional ad hoc reason why the rate constants inside and outside the cell are different. This additional difference is not necessary in the case where the reaction system is bistable. We now introduce several different cases of reaction networks and show the difference between cases which satisfy the requirements to be a metabolism and cases which do not.

## 2. Materials and Methods

We begin with a set of nine reactions involving six kinds of molecule. The molecules are labelled $A_{n}$ (*n* = 1,2,3,4 or 5) and W. Molecules $A_{1}$and $A_{2}\;$are designated as food molecules (equivalent to the food set in the RAF model). These are present at high concentration in the external medium as well as inside the cell. Molecules $A_{3}$, $A_{4}$ and $A_{5}$ are designated as biomolecules. They are present at a significant concentration inside the cell, and in low (or zero) concentration outside. Molecule W is a waste molecule formed by breakdown of the biomolecules. Molecule $A_{n}$ has mass *n* units and standard free energy of formation $\;∆G_{form}^{0}=\left(n-1\right)\times 0.2RT$. Molecule W has mass 1 and standard free energy of formation $\;∆G_{form}^{0}=-RT$.

The nine reactions are shown in Table 1. Reactions 1 to 4 are formation reactions of molecules $A_{2}$ to $A_{5}$ by progressive addition of$\;A_{1}$. Reactions 5 and 6 make cyclic autocatalytic processes involving $A_{3},\;\;A_{4}$ and $A_{5}$. Reactions 7 to 9 are breakdown reactions in which the catalysts decay to W. All reactions are reversible. The free energy change for the reaction, ∆G, (shown in Table 1) is the difference between the free energy of formation of the products and the reactants. The net reaction rate of reaction *i* is $r_{i}$ (where $C_{i}\;$denotes the concentration of$\;A_{i}$). In each case, the downhill reaction has a rate constant $u_{i}$ and the uphill reaction has a rate constant $u_{i}K$, with an equilibrium constant K that is less than 1 (except for reactions 5 and 6, which have K = 1).

**Figure 1:**
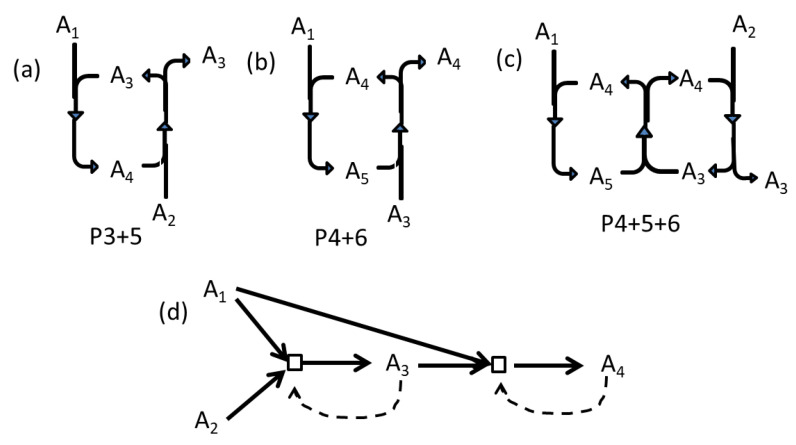
Autocatalytic processes can be formed by combining the reactions in Table 1, as described in the text. (**a**) Process P3 + 5 gives autocatalytic synthesis of $A_{3}$ from $A_{1}$ and $A_{2}$. (**b**) Process P4 + 6 gives autocatalytic synthesis of $A_{4}$ from $A_{1}$ and $A_{3}$. (**c**) Process P4 + 5 + 6 gives autocatalytic synthesis of $A_{3}$ from $A_{1}$ and $A_{2}$. (**d**) The reaction network drawn in the RAF manner, where squares indicate reactions and dashed arrows indicate catalytic effects.

There are several autocatalytic processes in this reaction network. The reason for including autocatalytic processes is the expectation that these can create a bistable reaction system that can maintain a difference between the concentrations inside and outside. Summing reactions 3 and 5 together gives process P3 + 5, which is autocatalytic formation of $A_{3}$ from $A_{1}$ and $A_{2}$. This is shown in Figure 1a, and can be written $A_{1}+A_{2}+A_{3}+A_{4}\to 2A_{3}+A_{4}$. If $A_{3}$ is already present, then this process can form more, even if the direct formation of $A_{3}$ without catalysis (reaction 2) does not occur. Similarly, summing reactions 4 and 6 together gives process P4 + 6, which is autocatalytic formation of $A_{4}$ from $A_{1}$ and $A_{3}$. This is shown in Figure 1b, and can be written $A_{1}+A_{3}+A_{4}+A_{5}\to 2A_{4}+A_{5}$. Summing reactions 4, 5 and 6 together gives process P4 + 5 + 6, which is autocatalytic formation of $A_{3}$. This is shown in Figure 1c, and can be written $A_{1}+A_{2}+A_{3}+2A_{4}+A_{5}\to 2A_{3}+2A_{4}+A_{5}$. Processes P4 + 5 and P4 + 5 + 6 work in absence of direct synthesis of $A_{3}$ and $A_{4}$ (reactions 2 and 3). Molecule $A_{5}$ is a participant in these cyclic processes; thus, it is automatically formed if $A_{3}$ and $A_{4}$ are formed.

Figure 1d shows the reaction network drawn in the RAF manner, where squares indicate reactions and dashed lines indicate catalytic effects. However, the mechanism of the reactions is not clear from Figure 1d, and the existence of $A_{5}$ is not apparent.

Autocatalytic processes become relevant when the corresponding direct synthesis reaction does not occur. Therefore, we consider the three cases shown in Table 2. Case 1 relies on P4 + 6 and P4 + 5 + 6 for formation of $A_{3}$ and $A_{4}$. The direct synthesis rate constants $u_{2}$ and $u_{3}$ are 0. Case 2 relies on P3 + 5. Processes P4 + 6 and P4 + 5 + 6 cannot occur in Case 2 because $u_{6}\;$= 0. We therefore need to include the direct synthesis of $A_{4}$ ($u_{3}$ = 1), but the direct synthesis of $A_{3}$ is still excluded ($u_{2}$ = 0). In Case 3, all the autocatalytic processes are eliminated ($u_{5}=u_{6}=0$) and the direct synthesis reactions 1–4 are all included. In all three cases, the rate constants for the breakdown reactions 7–9 are equal to v, and we calculate the behaviour of the reaction systems as a function of the variable rate v. In each of the cases, we have set the rate of the reactions that sustain the metabolism to 1. Reactions must also occur sometimes that break down catalytic molecules. By setting the rates of these reactions to v  we have a simple way of controlling the breakdown rates relative to the rates of the constructive reactions. Cases 1, 2 and 3 correspond to reaction systems that are autocatalytic and bistable, autocatalytic and monostable, and not autocatalytic, respectively. The results below demonstrate the differences between these cases. We show that Case 1 satisfies all the desired criteria for a metabolism, but some of these criteria are no longer satisfied in Cases 2 and 3.

## 3. Results

### 3.1. Case 1

Firstly, we calculate the concentrations outside the cell. We assume that $C_{1}\;$is maintained at $C_{1}^{out}$ = 1M, and that reaction 1 can occur freely, so that $C_{2}\;$is maintained in equilibrium with $C_{1}$; hence, $C_{2}^{out}=K{\left(C_{1}^{out}\right)}^{2}$ = 0.819 M. W is maintained at a fixed low concentration $W^{out}\;$= 0.01 M. If W were in equilibrium with $C_{1}$ then its concentration would be $W^{eq}=C_{1}^{out}\exp\left(+1\right)$ = 2.718 M. Thus, by fixing $W^{out}$ to be much less than $W^{eq}$, we ensure that the external environment is maintained out of equilibrium and that there is a driving force for the metabolic system inside the cell. The steady-state concentrations $C_{3},\;C_{4}\;$and $C_{5}$ are found by solution of the following differential equations using the fourth-order Runge-Kutta method until convergence is reached.

(1) $$\frac{dC_{3}}{dt}=r_{2}-r_{3}+2r_{5}-r_{6}-r_{7}$$

(2) $$\frac{dC_{4}}{dt}=r_{3}-r_{4}-r_{5}+2r_{6}-r_{8}$$

(3) $$\frac{dC_{5}}{dt}=r_{4}-r_{6}-r_{9}$$

The rates $r_{i}$ are given in Table 1. As a measure of the metabolic rate, we use the rate of mass flow from food molecules (1 and 2) to biomolecules (3–5), which is

(4) $$R_{met}=3r_{2}+r_{3}+r_{4}+2r_{5}\;.$$

In the steady state, this rate is balanced by an equal rate of mass flow of biomolecules decaying to waste (Equation (5)):

(5) $$R_{dec}=3r_{7}+4r_{8}+5r_{9}.$$

In order to keep the concentrations of food molecules and waste fixed at the external concentrations, there must be a supply rate of food molecules and a removal rate of W by an environmental recycling or replenishment process. In the steady state, all four rates are equal (Equation (6)).

(6) $$R_{supply}=R_{met}=R_{dec}=R_{remove}$$

As this reaction system is bistable, the steady-state concentrations depend on the initial conditions. We consider two initial conditions, called low-concentration (LC) and high-concentration (HC) initial conditions. For the LC conditions, we start with $C_{3}=C_{4}=C_{5}=10^{-4}$ M. For the HC conditions, we set the concentrations to $C_{n}={\left(C_{1}^{out}\right)}^{n}K^{n-1}$, which is the concentration that would occur if the molecules were in equilibrium with $C_{1}$ and there were no decay to W (i.e., if v were zero).

**Figure 2:**
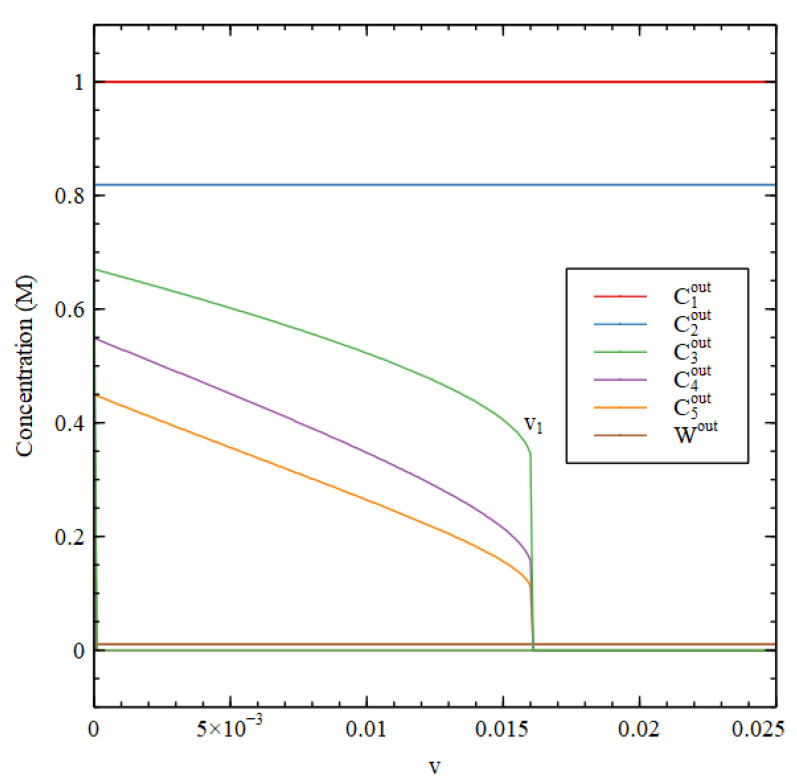
Steady-state concentrations $C_{n}^{out}$ outside the cell as a function of the decay rate constant v for Case 1. For $v>v_{1}$ only the LC solution is stable. For $v<v_{1}$, both HC and LC solutions are stable.

Figure 2 shows the steady-state concentrations that arise as a function of v, starting from the two initial conditions. There is a transition point at $v_{1}$ = 0.016. For $v<v_{1}$, there are two stable solutions—the HC solution with high concentrations of the three biomolecules and the LC solutions where the biomolecule concentrations are very small. Starting from HC initial conditions gives the HC solution, and starting from LC initial conditions gives the LC solution. For $v>v_{1}$, only the LC solution is stable, and this solution is reached starting from both initial conditions.

We now consider concentrations inside the cell. Initially, we consider the cell to be a small compartment of fixed volume which does not grow. Later, we will consider cells with membranes that can grow and divide. For, the fixed-volume compartment, the cell boundary is permeable to $A_{1},\;A_{2}$ and W, with inflow and outflow proportional to rate q, and it is impermeable to $A_{3},\;A_{4}$ and $A_{5}$. Since $A_{1},\;A_{2}$ and W have variable concentration inside the cell, we need three additional differential equations for these molecules.

(7) $$\frac{dC_{1}}{dt}=q\left(C_{1}^{out}-C_{1}\right)-2r_{1}-r_{2}-r_{3}-r_{4}$$

(8) $$\frac{dC_{2}}{dt}=q\left(C_{2}^{out}-C_{2}\right)+r_{1}-r_{2}-r_{5}$$

(9) $$\frac{dW}{dt}=q\left(W^{out}-W\right)+3r_{7}+4r_{8}+5r_{9}$$

The Equations (1)–(3) for $C_{3},\;C_{4}$ and $C_{5}$ are the same as outside the cell (although the concentrations will be different). $R_{met}$ and $R_{dec}$ are calculated as before. Instead of the supply of food and removal of waste via environmental recycling, we now have diffusion of food into the cell and diffusion of waste out.

(10) $$R_{in}=q\left(C_{1}^{out}+2C_{2}^{out}-C_{1}-2C_{2}\right)$$

(11) $$R_{out}=q\left(W-W^{out}\right)$$

In the steady state, the mass flow rate at all the steps is equal.

(12) $$R_{in}=R_{met}=R_{dec}=R_{out}$$

**Figure 3:**
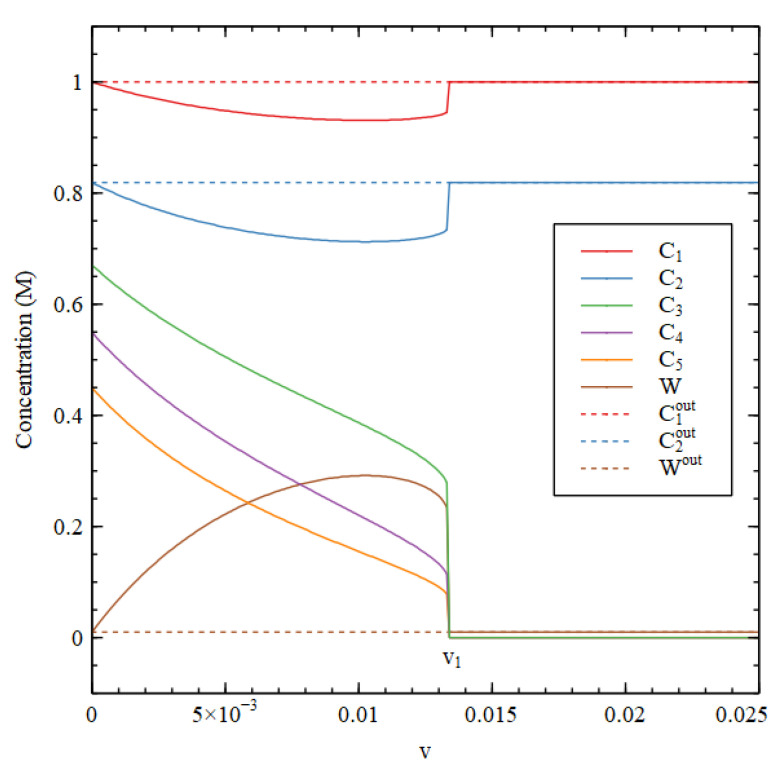
Steady-state concentrations inside ($C_{n}$) and outside ($C_{n}^{out}$) the cell as a function of decay rate v for Case 1.

Figure 3 compares steady-state concentrations inside and outside the cell. We first determine the external concentrations, starting from the LC initial conditions. Then, keeping the external concentrations fixed in their steady state, we determine the steady state inside the cell by numerical solution of the differential equations for the inside, starting from the HC initial conditions. We want to show that the HC solution inside the cell can be stable when the outside is in the LC state.

There is an HC solution inside the cell that is stable up to a transition value $v_{1}$, which is slightly lower than the $v_{1}$ in Figure 2. It is apparent that the waste concentration W inside the cell is fairly high, whereas it is fixed at a low concentration outside. When the inside is in the HC state, $C_{1}$ and $C_{2}$ are lower inside than out because there has to be a net flow of food molecules inwards. The external concentrations $C_{3}^{out},\;C_{4}^{out}$and $C_{5}^{out}$ are very close to zero and are not shown in Figure 3.

**Figure 4:**
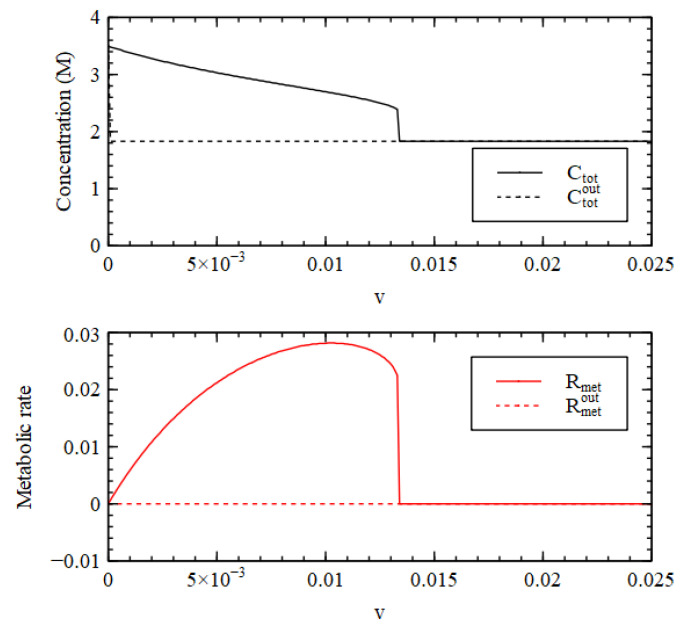
Total concentration inside ($C_{tot}$) and outside ($C_{tot}^{out})$ the cell and metabolic rate inside ($R_{met})\;$and outside ($R_{met}^{out}$) as a function of decay rate v for Case 1.

It is useful to look at the total concentration, shown in Figure 4. For$\;v<v_{1}$, the total concentration is higher inside than out, hence there is a positive osmotic pressure inside the cell, as we require for Criterion 2. We will show in a later section of this paper that when this is the case, the osmotic pressure can drive growth and division of lipid vesicles. However, firstly, we want to establish under what conditions the reaction system can maintain a positive osmotic pressure in a compartment of fixed volume. For $v>v_{1}$, the total concentration inside collapses to the concentration outside, so the osmotic pressure is lost.

A similar result is seen in Figure 4 for the metabolic rate $R_{met}$ (Equation (4)). The external metabolic rate is very close to zero when the outside is in the LC state because the rate constants $u_{2}$ and $u_{3}$ are zero, and because the concentrations of the catalysts are very low, so the rates of the reactions in the catalytic processes are also very low. Thus, when $\;v<v_{1}$, $R_{met}$ in the HC state inside the cell is much higher than $R_{met}^{out}$ outside the cell, as we require for Criterion 3. Thus, all three criteria are satisfied for $v<v_{1}$, when the reaction system is bistable.

### 3.2. Case 2

**Figure 5:**
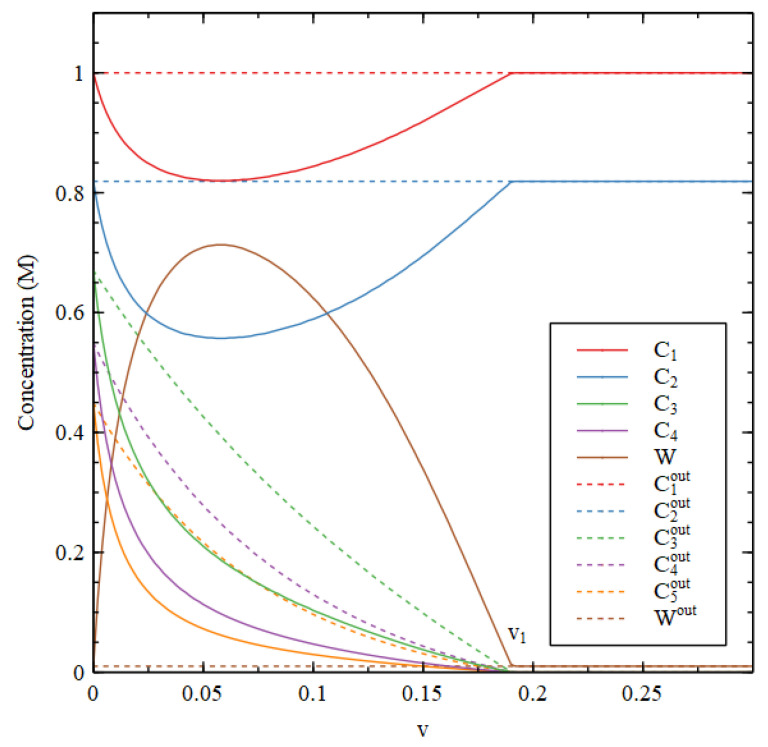
Steady-state concentrations inside ($C_{n}$) and outside ($C_{n}^{out}$) the cell as a function of decay rate v for Case 2.

We now consider Case 2, in which process P3 + 5 is permitted (by setting $u_{3}$ = 1), and where processes P4 + 6 and P4 + 5 + 6 are prevented (by setting $u_{6}$ = 0). This case still relies on an autocatalytic cycle to synthesize $A_{3}$, because the direct reaction 2 is not possible ($u_{2}$ = 0). Case 2 is monostable, therefore it does not matter whether we start with LC or HC initial conditions. Figure 5 shows the stable state concentrations inside and out. There is a transition point at $v_{1}$ = 0.192. For $v<v_{1}$, there is an HC solution with high $C_{3},C_{4}$ and $C_{5}$, whereas for $v>v_{1}$, these concentrations fall very close to zero and the concentrations $C_{1},\;C_{2}$ and W become equal to their external values.

**Figure 6:**
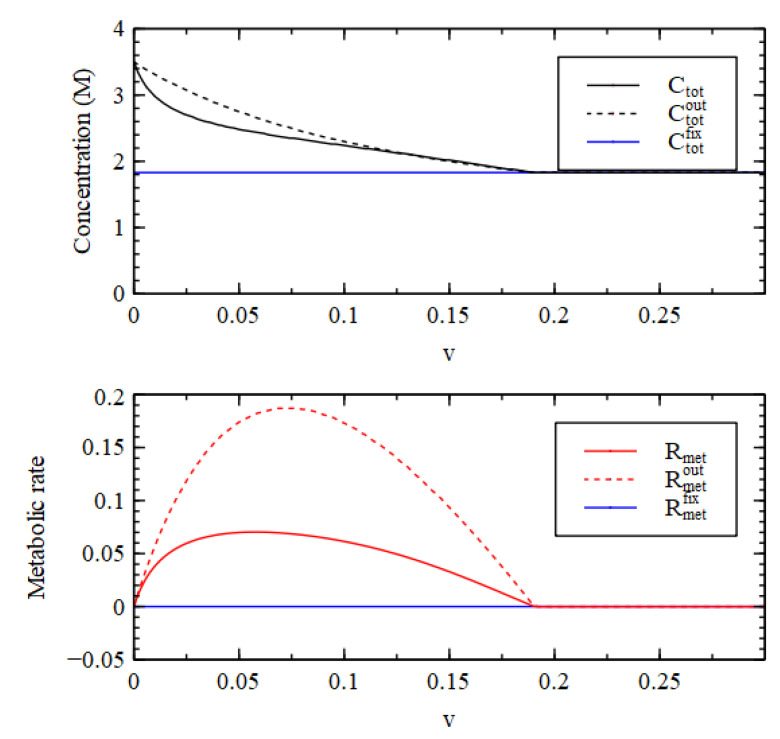
Total concentration and metabolic rate for Case 2, shown inside the cell ($C_{tot}.\;R_{met}$), outside the cell when v is the same inside and out ($C_{tot}^{out},\;R_{met}^{out}$), and outside the cell when the external v is fixed at 0.3 ($C_{tot}^{fix},\;R_{met}^{fix}$).

In Case 2, biomolecules can survive at high concentration for much larger decay rates than in Case 1. Nevertheless, Case 2 is not a good model for a metabolism as it stands because all the molecules with the exception of W have a higher concentration outside than inside. Figure 6 shows that the total concentration inside is less than outside and the metabolic rate inside is less than outside. Thus, Case 2 does not satisfy Criteria 2 and 3. This shows that the existence of an autocatalytic process in the reaction system (P3 + 5 in this case) and the presence of biomolecules that are maintained at a high concentration by this process is not sufficient for the reaction system to satisfy the requirements for a metabolism.

The reason for the failure of Case 2 is that there is no stable LC state on the outside. We could in principle begin with with zero concentration of $A_{3}$ outside, and we could argue that $A_{3}$ would never form because $u_{2}$ = 0. However, in a real case, there would always be some possibility of leakage of $A_{3}$ from the inside, either via a small rate of permeability through the membrane or due to occasional bursting of cells. There is also a very small rate of formation of $A_{3}$ from W, via the reverse of reaction 7. Thus, we are bound to have a small initial concentration of these molecules by one means or another, and if the LC state is not stable, then the concentrations of $A_{3},\;A_{4}\;$and $A_{5}$ will rise to high values. This rules out Case 2 as a model for metabolism as it stands.

However, we can rescue Case 2 if we are prepared to make the assumption that the decay rate v of the biomolecules is larger on the outside than the inside. (We will discuss further below why this might be the case). We fixed the external decay rate to be $v_{out}$ = 0.3, which is higher than $v_{1}$, and the decay rate v inside was allowed to vary as before. The concentrations inside the cell are the same as before, but $C_{3},\;C_{4}$ and $C_{5}$ are now close to zero outside the cell because $v_{out}>v_{1}$. Figure 6 shows that when $v_{out}$ is fixed at a high rate, the total external concentration $C_{tot}^{fix}$ and the external metabolic rate $R_{met}^{fix}$ are both lower than the internal values ($C_{tot}$ and $R_{met}$).

Thus, with the additional assumption that the decay rate is higher outside than in, Case 2 now satisfies the required criteria for a metabolism. It is possible to think of reasons why this might be true in a real case. The decay reactions 7–9 might be driven by UV light, and being inside the compartment might provide some protection from this, so v would be larger outside than inside. Or possibly, the breakdown reactions could be driven by interaction with some additional molecule that is not permeable to the membrane, so the breakdown would occur outside but not inside. Although these reasons seem possible, they are somewhat ad hoc, in that an extra condition must be added that was not part of the definition of the reaction network. The actual degree of UV protection afforded by a lipid membrane may not be very large, and what impermeable molecule might be that would cause breakdown outside is unclear. Other reasons for the difference between inside and outside could be proposed if desired. However, if any of these mechanisms were true, we would have the rather odd situation that life would exist inside the cell because of the absence of an inhibiting effect that occurs outside the cell, rather than because of the presence of something constructive inside the cell. Life would be just waiting to pop up as soon as the inhibiting factor is removed! This does not fit with our intuition that the origin of life is an unlikely event that requires the coming together of favourable circumstances.

An alternative way to rescue Case 2, rather than making v larger on the outside, would be to make the reactions of the autocatalytic processes ($u_{3},\;u_{4},\;u_{5}$) small or zero on the outside. This would obviously make the model work, because the $C_{3},\;C_{4}\;$and $C_{5}$ would remain low outside, but again it is ad hoc. It is not obvious why the chemistry should be different outside the cell from inside. Piedrafita et al. [30] have studied two reaction networks which they call protometabolism 1 and 2—PM1 and PM2. PM2 is bistable, with similar properties to our Case 1, and PM1 is monostable, with similar properties to our Case 2. These authors did not look at the reactions outside the cell, and simply assumed that these reactions did not occur. This requires a reason why the reaction rates for the autocatalytic processes (analogous to our $u_{3},u_{4},\;u_{5}$) should be zero outside. This point was not considered by Piedrafita et al. [30]. We consider this issue to be important, because if the rate constants are equal inside and out and if the system has only one stable solution with high biomolecule concentration, the reaction system will not satisfy the criteria for a metabolism.

### 3.3. Case 3

So far, we have shown that in bistable reaction systems, there is a natural explanation of why the difference in total concentration and metabolic rate between the inside and the outside is maintained, whereas in monostable systems, these differences are only maintained if there is an extra ad hoc reason why reaction rate constants are different on the inside and outside. We now take this argument one step further, by showing that if we do allow rate constants to be different inside and out, then we no longer need there to be any autocatalytic processes at all.

**Figure 7:**
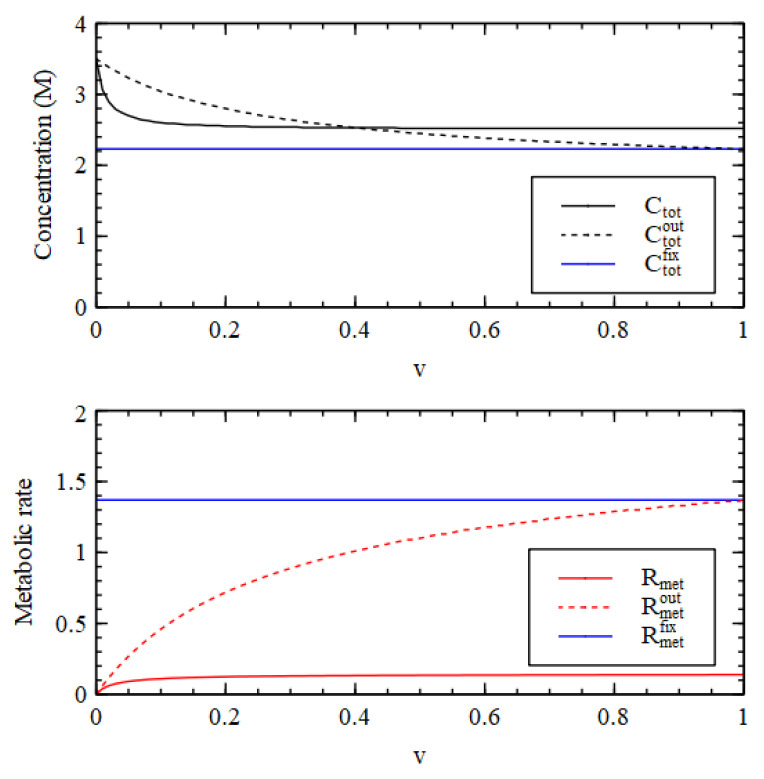
Total concentration and metabolic rate for Case 3, shown inside the cell ($C_{tot}.\;R_{met}$), outside the cell when v is the same inside and out ($C_{tot}^{out},\;R_{met}^{out}$), and outside the cell when the external v is fixed at 1.0 ($C_{tot}^{fix},\;R_{met}^{fix}$).

In Case 3, all the direct reactions 1–4 occur with u=1, and none of the autocatalytic processes occur, because $u_{5}=u_{6}=0$. In this case, there is only one solution. The concentrations $C_{3},\;C_{4}\;$and $C_{5}$ reach high values both inside and outside, that decrease slowly with v, and there is no transtion point. The total concentration is lower inside than out at small v, but becomes higher inside for high v, mostly because of the accumulation of W (Figure 7). However, the external metabolic rate is always much higher than the internal rate. It should be remembered that these metabolic rates are the rates of mass conversion from food molecules to biomolecules per unit volume. Given that the external environment has a much larger volume than the cell, this means huge amounts of food molecules must be supplied, which seems unreasonable.

Now we try to rescue Case 3 by fixing the external decay rate to be a higher rate than inside. If $v_{out}$ is fixed at 1.0, then the total exterior concentration becomes $C_{tot}^{fix}$, shown in Figure 7, which is now lower than the interior concentration $C_{tot}$ for the whole range of v. However, the external metabolic rate becomes $R_{met}^{fix}$, which is very high. So this still does not satisfy Criterion 3.

We can force Case 3 to satisfy all the criteria, if we simply say that the synthesis reactions of the biomolecules do not occur outside the cell ($u_{2}=u_{3}=u_{4}=0).$ In this case only $C_{1},\;C_{2}$ and W exist outside the cell, and $R_{met}^{out}$ is fixed at zero, trivially, so Criterion 3 is also satisfied. Our point here is two-fold. Firstly, Case 3 seems to be too simple to be a good model for a metabolism, as it only works if we prohibit the direct synthesis reactions from occuring outside while allowing them to happen inside, which seems artificial. Secondly, however, if there really is a good reason why these reactions should be different inside and out, then this case shows that all three requirements for the existence of a metabolism can be satisfied with a reaction network having no autocatalytic processes at all.

### 3.4. Cell Growth and Division

So far, we considered only compartments of fixed volume. In this section, we will consider lipid vesicle compartments in which growth and division are possible. The mechanism for growth and division used here is very similar to that introduced in references [28,29,30].

Experiments often use vesicles with a radius of approximately 50 nm [31,32]. We will therefore define a standard vesicle of this size, and measure sizes of growing vesicles relative to this standard size. The radius, suface area and volume of a standard vesicle are $r_{st}=5\times 10^{-8}m$, $S_{st}=4\pi r_{st}^{2}$, and $V_{st}=4\pi r_{st}^{3}/3$.

If a vesicle has a volume V, then its surface area, if it were a sphere, would be $S_{sph}={\left(36\pi V^{2}\right)}^{1/3}$. However, the actual surface area of the vesicle also depends on the amount of lipid in the membrane. If L is the number of lipid molecules in the membrane, and $a_{L}$ is the surface area per lipid in a relaxed membrane, then the natural surface area, if it is a relaxed state, is $S_{L}=a_{L}L/2$, where the factor of 2 occurs because of the two sides of the lipid bilayer. The actual surface area S is the larger of $S_{sph}$ and $S_{L}$. When $S=S_{L}$ the vesicle is an irregular, non-spherical shape because its surface area is larger than the minimum area needed to enclose a sphere of volume V.  When $S=S_{sph}$, the vesicle is spherical, and the membrane is under tension, because its surface area is larger than the natural surface area of a relaxed membrane with L lipid molecules.

The tension is proportional to the area strain ε, which is the relative increase in surface area above the natural area: $\varepsilon =\left(S/S_{L}-1\right)$. This results in a pressure inside the vesicle proportional to ε/r. If the concentrations of solutes inside and outside the vesicle are different, then there is an osmotic pressure difference $∆\Pi =RT\left(C_{tot}-C_{tot}^{out}\right)$. For simplicity, we have assumed that all the solutes behave like ideal gases, so the osmotic pressure is directly proportional to the concentration. The balance of osmotic pressure and membrane tension results in the following equation for the growth of the volume.

(13) $$\frac{dV}{dt}=gV_{st}\frac{S}{S_{st}}\left(C_{tot}-C_{tot}^{out}-\frac{\tau r_{st}}{r}\left(\frac{S}{S_{L}}-1\right)\right)$$

For convenience, we have incorporated the RT factor into the definition of constants τ and g. A factor of $r_{st}\;$has been added, so r is measured relative to the standard vesicle, and the constant τ for the membrane tension has units of concentration (molar). The volume increase is due to water passing through the membrane, therefor the rate scales in proportion to the surface area. A factor of $V_{st}\;$has been added to give a volume scale appropriate to the standard size vesicle. The time scale for growth is determined by g, which has units of time^−1^molar^−1^. In the examples below, we have g=1, and τ=10.

If there is a fixed amount of lipid in the membrane, and a positive osmotic pressure, the volume will grow until the osmotic pressure is balanced by the membrane tension. There is a maximum area strain $\varepsilon_{c}$ of approximately 0.1 that can be maintained by the membrane. A vesicle will burst and release some of its contents if ε exceeds $\varepsilon_{c}$ [33,34]. Repeated cycles of swelling and bursting can be followed in experiments [35]. However, if the osmotic pressure is not too large, bursting will not occur. In this paper, we assume that the membrane is strong enough to maintain the pressure generated by the internal metabolic reactions without bursting.

If lipid is available in the solution outside and inside the membrane, then transfer of lipid molecules into and out of the membrane is possible, so the membrane surface area $S_{L}$ can grow at the same time the volume is increasing. Lipids are only sparingly soluble, and above a critical concentration $C^{*}$, lipid bilayers will spontaneously form, until the concentration falls to $C^{*}$. The number of lipid molecules on each surface of the bilayer is $S_{L}/a_{L}$. The rate of motion of lipid molecules into and out of the membrane on the two sides is $\frac{S_{L}}{a_{L}}\left(k_{in}(C_{L}+C_{L}^{out}\right)-2k_{out})$, where $C_{L}$ and $C_{L}^{out}$ are the lipid concentrations inside and outside the vesicle, and $k_{in}$ and $k_{out}$ are the rate constants for molecules entering and leaving the membrane. When the solution concentration is $C^{*}$, the membrane is in equilibrium with the solution. This means that $k_{in}=k_{out}/C^{*}$. We assume in what follows that the external lipid concentration is fixed at $C^{*},$ and the internal concentration will also tend to $C^{*}$ if the membrane is relaxed. A relaxed vesicle will not increase in membrane area if the solution on both sides of the membrane has concentration $C^{*}$. However, it is observed experimentally [31] that transfer of lipids from relaxed vesicles to osmotically swollen vesicles occurs. This means that swollen vesicles gain lipids at solution concentrations where relaxed vesicles do not grow. To account for this, we assume that the rate of lipids entering the membrane increases when the membrane is under tension with input rate proportional to $S/S_{L}$, whereas the rate of lipids leaving the membrane remains unchanged when the membrane is under tension. The rate of change of the number of membrane lipids for a membrane under tension can therefore be written $\frac{S_{L}}{a_{L}}\left(\frac{k_{out}}{C^{*}}\left(C_{L}+C_{L}^{out}\right)\frac{S}{S_{L}}-2k_{out}\right)$. The rate of change of lipid surface area, assuming lipids flip between bilayers rapidly to keep equal numbers of molecules on both surfaces, is $a_{L}/2$ times the rate of exchange of molecules. Hence, finally:

(14) $$\frac{dS_{L}}{dt}=k_{out}S_{L}\left(\frac{\left(C_{L}+C_{L}^{out}\right)}{2C^{*}}\frac{S}{S_{L}}-1\right)$$

The rate constant $k_{out}$ is set to 1 in the examples below, which sets the timescale for membrane area growth comparable to that for vesicle volume growth.

Equation (14) says that the membrane area will grow either if the vesicle is under tension, or if the vesicle is relaxed but the internal concentration is higher than $C^{*}$. We therefore introduce further reactions into the system to allow for the possibility that the metabolism drives lipid synthesis inside the vesicle. A precursor molecule $L_{1}\;$is assumed to be present in the external environment, and combination of $L_{1}$ with $A_{1}$ yields the lipid L. However, this direct reaction does not occur, but it is driven in two steps by a catalytic process involving $A_{3}$, as shown in Table 3. Thus, lipid synthesis will only occur when the metabolism is operating and maintaining a high concentration of $A_{3}$. We assume that the free energies of formation of the precursor $L_{1}$, the intermediate $L_{2}$ and the final lipid L are 0.4RT, RT, and 0.8RT, respectively. The free energies of the reactions are given in Table 3.

As the volume is changing with time, it is easier to deal with the number of molecules in the vesicle, $M_{i}$, and then to determine the concentrations as $C_{i}=M_{i}/\left(N_{A}V\right)$, where V is in litres, $C_{i}$ is in moles/litre and $N_{A}$ is Avogadro’s number $=6.02\times 10^{23}$. Equations (15)–(23) for the molecule numbers need to be solved concurrently with 13 and 14 for the volume and lipid surface area.

(15) $$\frac{dM_{1}}{dt}=N_{A}V_{st}\frac{qS}{S_{st}}\left(C_{1}^{out}-\frac{M_{1}}{N_{A}V}\right)-N_{A}V\left(2r_{1}+r_{2}+r_{3}+r_{4}+r_{11}\right)$$

(16) $$\frac{dM_{2}}{dt}=N_{A}V_{st}\frac{qS}{S_{st}}\left(C_{2}^{out}-\frac{M_{2}}{N_{A}V}\right)+N_{A}V(r_{1}-r_{2}-r_{5})$$

(17) $$\frac{dM_{3}}{dt}=N_{A}V(r_{2}-r_{3}+2r_{5}-r_{6}-r_{7}-r_{10}+r_{11})$$

(18) $$\frac{dM_{4}}{dt}=N_{A}V\left(r_{3}-r_{4}-r_{5}+2r_{6}-r_{8}\right)$$

(19) $$\frac{dM_{5}}{dt}=N_{A}V(r_{4}-r_{6}-r_{9})$$

(20) $$\frac{dM_{W}}{dt}=N_{A}V_{st}\frac{qS}{S_{st}}\left(W^{out}-\frac{M_{W}}{N_{A}V}\right)+N_{A}V(3r_{7}+4r_{8}+5r_{9})$$

(21) $$\frac{dM_{L1}}{dt}=N_{A}V_{st}\frac{qS}{S_{st}}\left(L_{1}^{out}-\frac{M_{L1}}{N_{A}V}\right)-N_{A}Vr_{10}$$

(22) $$\frac{dM_{L2}}{dt}=N_{A}V\left(r_{10}-r_{11}\right)$$

(23) $$\frac{dM_{L}}{dt}=\frac{k_{out}S_{L}}{a_{L}}\left(\frac{M_{L}}{N_{A}VC^{*}}-1\right)+N_{A}Vr_{11}$$

We have assumed that the membrane is permeable to the precursor $L_{1}$, but not to the intermediate $L_{2}$. The membrane is not permeable to the lipid L itself, but L can cross between the two sides by entering and leaving the membrane. In calculating the permeability terms above, we assumed that the constant q in Equations (7)–(9) for the fixed-volume compartment applies to a compartment with volume $V_{st}$ and surface area $S_{st}$. If the rate of concentration change in the standard vesicle is q, the rate of change of the number of molecules is $N_{A}V_{st}q.$ The rate of change in the vesicle of variable size increases in proportion to *S*, which gives a rate constant $N_{A}V_{st}\frac{qS}{S_{st}}\;$in the equations above.

**Figure 8:**
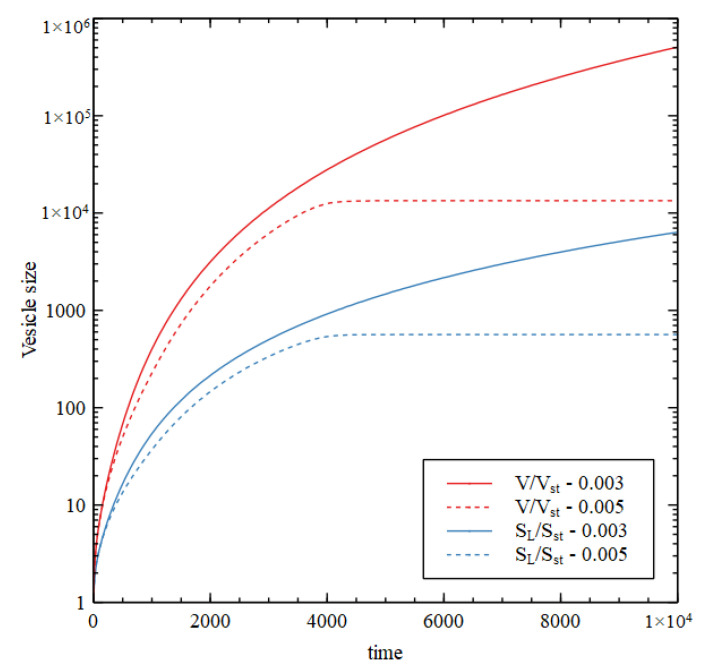
Ratio of the vesicle volume to standard vesicle volume ($V/V_{st}$) and ratio of lipid surface area to standard vesicle surface ($S_{L}/S_{st}$) as a function of time for Case 1 reaction network with no internal lipid synthesis ($u_{L}=0),\;$for two values of the decay rate, v  = 0.003 and 0.005.

We suppose that the critical lipid concentration is $C^{*}=4\times 10^{-3}$ M, which is appropriate for fatty acids, and the external concentration is fixed at $C_{L}^{out}=C^{*}$. The external concentrations of the precursor and intermediate are $C_{L1}^{out}=0.1\;$ M, and $C_{L2}^{out}=0.0\;$M. Figure 8 shows results using Case 1 reaction rates, assuming that L and $L_{1}$ are present, but the rate constants for reaction 10 and 11 are $u_{L}=0,\;$so there is no lipid synthesis by the metabolism. The inside begins with HC initial conditions while the outside is in the LC state. Therefore, there is a positive osmotic pressure that drives increase in volume. The membrane is then under tension ($S>S_{L})$, so $S_{L}$ increases due to addition of lipid molecules from the outside. Continued growth is seen at v=0.003,  but at v=0.005  the internal concentrations collapse to the external concentration after a time, so there is no further growth.

**Figure 9:**
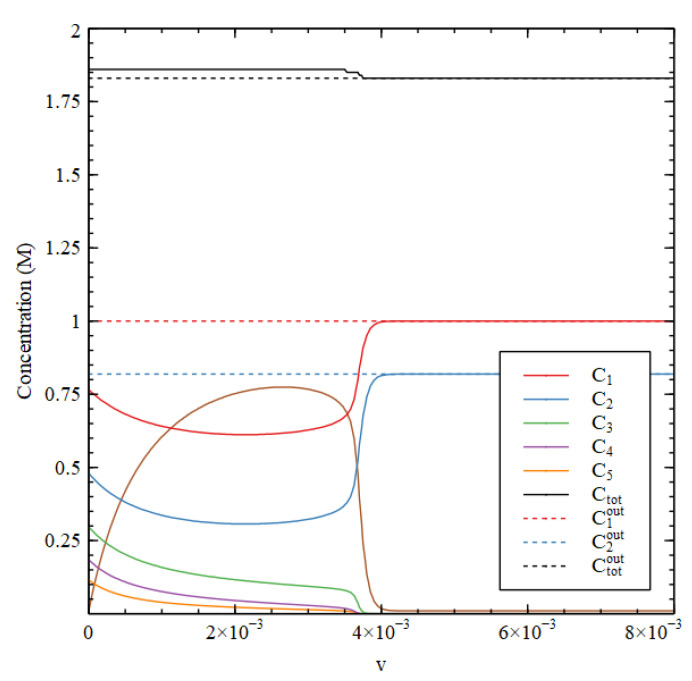
Concentrations inside the cell ($C_{n}$) after time 10^4^ compared with stationary external concentrations ($C_{n}^{out}$), shown as a function of decay rate v for Case 1 reaction network with no internal lipid synthesis.

Figure 9 shows the concentrations inside the cell at the end of the simulation run (time 10000). There is a transition point at $v_{1}\approx 0.004$. For $v<v_{1}$ the osmotic pressure difference is maintained by the metabolism, and the volume and lipid surface area grow indefinitely. If $v>v_{1}$ the osmotic pressure difference is not maintained and the vesicle stops growing. For $v<v_{1}$, all three criteria for a metabolism are satisfied while the vesicle is growing continuously.

When lipid synthesis occurs inside the vesicle, we need to consider the possibility of vesicle division. As has been shown previously [27,28,29,30], a spherical vesicle cannot divide without losing contents because the membrane area needed to enclose a sphere of volume V is less than the membrane area needed to enclose two spheres of volume $\frac{V}{2}$. Thus, if a vesicle is growing with a membrane under tension, as in Figure 8, then it cannot divide. For division to occur we need the membrane to be relaxed, i.e., the vesicle must be non-spherical, with a lipid surface area $S_{L}\;$ greater than the minimal surface area required for the sphere of the current volume $S_{sph}\left(V\right)$. After division, the minimal surface area for the two smaller spheres is $2S_{sph}\left(\frac{V}{2}\right)=2^{\frac{1}{3}}S_{sph}\left(V\right)S\;Sph\;ivision,\;the\;minimal\;surface\;area\;for\;the\;two\;smaller\;spheres\;is$revious e area of a sphere of volume. Thus, division of the original vesicle can occur only if $S_{L}>2^{1/3}S_{sph}\left(V\right).$

To achieve vesicle division at the same time as the osmotic pressure is driving volume increase, we need the surface area to grow proportionately faster than the volume. This means that the lipid concentration must be higher than $C^{*}$, so that there is a net addition of molecules to the relaxed membrane. The exterior concentration can be temporarily higher than $C^{*}$ if there is a sudden addition of lipid to the external medium. It is known that sudden addition of lipid micelles to vesicles causes vesicle division [32,36,37]. On the other hand, if the external concentration remains at $C^{*}$, the internal concentration can become higher than $C^{*}$ if there is lipid synthesis inside the vesicle.

**Figure 10:**
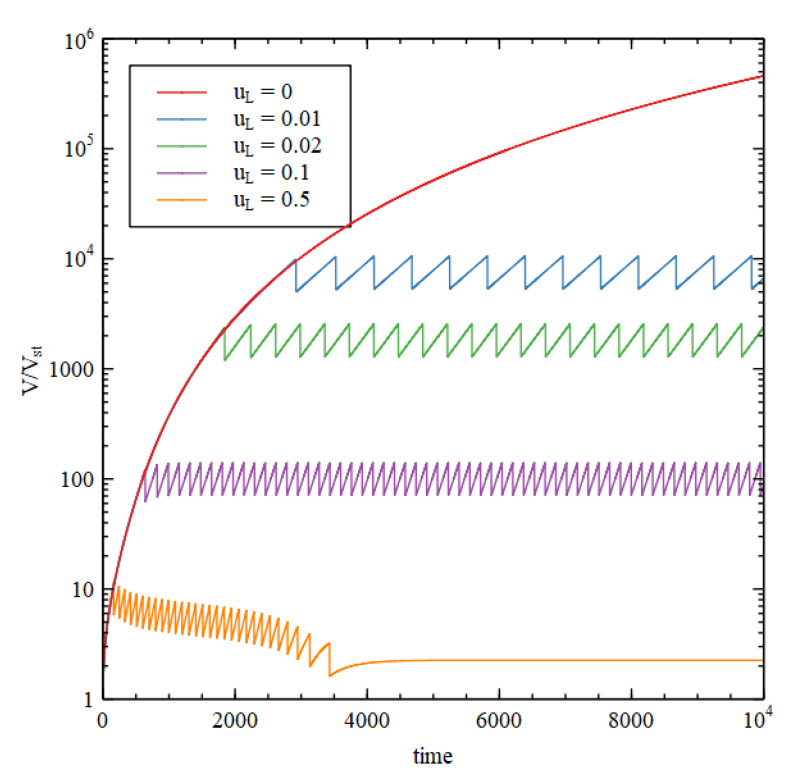
Case 1 reaction system in vesicle compartments with internal synthesis of lipid. Volume reltive to standard volume ($V/V_{st}$) is shown as a function of time for five different values of the lipid synthesis rate $u_{L}$. The decay rate is v=0.003  in all cases.

We now consider the case of internal lipid synthesis driven by the metabolism. We consider Case 1 with v=0.003, which is able to support continued vesicle growth when there is no internal lipid synthesis (as in Figure 8). We then add the lipid synthesis reactions 10 and 11, with rate constant $u_{L}$. We begin with one vesicle of standard size with internal concentrations equal to the HC initial conditions. Whenever the lipid surface area becomes large enough to satisfy the division condition,$\;S_{L}>2^{1/3}S_{sph}\left(V\right),$ the vesicle is divided into two half-sized vesicles, and we continue to follow the growth of one of these. Figure 10 shows the volume as a function of time for five values of $u_{L}$. For $u_{L}=0$, we have continued growth without division, as before. For $u_{L}>0$, we have repeated growth and division. The time required for division and the size at which division occurs decrease with increasing lipid synthesis rate. Interestingly, it is possible for lipid synthesis to be too fast. For the highest $u_{L}$ considered, the vesicle gets smaller on each division, because the division condition is reached before the size has doubled. Smaller vesicles have a larger surface area to volume ratio, so when the vesicle gets smaller, the permeability terms become larger relative to the internal reaction terms, and the difference between the internal and external concentrations cannot be maintained. Hence, growth and division stop after a few cycles.

In the case where continued growth occurs without division, as the growth is driven by autocatalytic reactions, we might expect that volume would grow exponentially with time. However, this is not so, because the rate of growth is limited by the rate of supply of food molecules. The vesicle settles into a state of steady growth where the internal concentrations are approximately constant in time and there is an approximately constant osmotic pressure. The membrane is only slightly swollen, and the tension term in equation 13 is small in comparison to the osmotic pressure. In this case the rate of volume increase is roughly proportional to the surface area. The rate of import of food molecules is also proportional to surface area, as is required if the internal concentration is constant. We thus have $\frac{dV}{dt}\sim V^{2/3}$, hence V increases in proportion to $t^{3}$, not exponentially with time. However, in the case where repeated cell division occurs, the time required for each division is constant. The number of vesicles doubles at each division; hence, the number of vesicles increases exponentially with time. The time per division is only a constant when there is balanced growth of the membrane and the vesicle contents, i.e., the time for doubling of the vesicle volume is equal to the time for doubling of the membrane area (more details in [28]). These doubling times are dependent on vesicle size, and it can be seen that the vesicle naturally tends to a size when they are equal. In this self-reproducing state, each subsequent generation has the same size and composition as the previous one.

In this section, we have considered the Case 1 reaction network coupled to lipid synthesis and shown that it can drive vesicle growth and division. Any of the other models could also be coupled to lipid synthesis in the same way, so that in all the cases where the criteria for a metabolism are satisfied in a fixed-volume compartment, the addition of internal lipid synthesis will lead to growth and division in a lipid vesicle.

### 3.5. RNA Synthesis

The assumption of metabolism-first theories is that it is possible to have a self-sustaining small-molecule metabolic system without the presence of information-carrying polymers such as RNA. The previous examples have all focused on this case, showing that it is possible in theory, provided the reactions system satisfies the three criteria. If a small-molecule metabolism did get going in a protocell, it could later begin to synthesize nucleotides by some process that is coupled to the metabolism (in the way that the lipid synthesis was coupled to metabolism in the previous section). Polymerization of nucleotides could then arise in a protocell that already had an established metabolism.

However, there is still little experimental evidence for a self-sustaining small-molecule metabolism, so it is still possible to argue for replication-first. In this section, we make the point that template-directed synthesis of RNA is inherently autocatalytic because RNA strands are required to synthesize more strands. This means that the RNA polymerization process itself can produce a reaction system that satisfies the criteria for a metabolism. RNA World theories obviously require a means of synthesis of nucleotides before polymerization of nucleotides into RNA strands can occur. However, there is no requirement that the reaction pathways that generate nucleotides should be autocatalytic or that they should satisfy the criteria for a metabolism. This section will show a clear example where nucleotides are synthesized by a direct reaction pathway that is not autocatalytic and which does not by itself satisfy the metabolism criteria. Polymerization then occurs autocatalytically, and the metabolism requirements are satisfied due to the polymerization reactions, not the small-molecule reactions.

In this section, we identify molecule $A_{5}$ as a nucleotide, and we call it N hereafter. Reactions 1–4 represent steps in the pathway synthesizing nucleotides. We assume that $u_{i}$ = 1 for all these direct reactions, and that the small-molecule autocatalytic processes do not occur (as in Case 3). We already showed above that Case 3 does not satisfy the metabolism criteria, unless we set $u_{i}$ to be zero for the formation reactions outside the cell. Here, on the contrary, reactions 1–4 occur freely both inside and out. So the nucleotide-synthesis pathway does not constitute a metabolism by itself, and cannot support cell growth and division. We then suppose that nucleotides can polymerize by a reaction N→P, where P represents a nucleotide that is part of an RNA polymer. Polymerization will generate many different sequences of many different lengths, and incorporation of all these things is beyond the scope of the present model. However, the simple N→P reaction captures the essence of the process. The polymerization rate is

(24) $$r_{pol}=\left(s_{P}+r_{P}P+k_{P}P^{2}\right)N\left(1-\frac{P}{P^{*}}\right),$$

where $s_{P},\;r_{P}$ and $k_{P}$ are rates of spontaneous polymerization (independent of current strand concentration), non-enzymatic template-directed synthesis (proportional to template concentration P), and ribozyme-catalyzed synthesis (proportional to both template and catalyst concentration—hence $P^{2}$). All three synthesis processes are proportional to nucleotide concentration N, and they are limited by some saturation of resources or space when P reaches a maximum concentration $P^{*}$. Inclusion of $P^{*}$ is necessary to prevent a runaway increase in polymer concentration. Cleavage reactions convert polymeric nucleotides back to single nucleotides P→X. We assume that the nucleotide X produced by cleavage is not the same as the nucleotide N prior to polymerization. For example, N could be an activated nucleotide, whereas X is not. We assume X cannot repolymerize, hence it is a waste molecule. The cleavage reaction occurs at rate aP. We ignore the other waste molecule W, because it is not important for the point we are making in this section. The decay reactions 7–9 do not occur in this example. Treating X as a waste molecule is the least favourable case for establishing polymerization, in comparison to cases where cleavage directly reforms N, or where recycling of X back to N is possible.

When $k_{P}$ is high and $s_{P}$ and $r_{P}$ are both low, RNA polymerization is bistable. In earlier papers [38,39,40,41] we have shown that this bistability results in two states that we called living (high polymer concentration with RNA synthesis dominated by the catalyzed process), and non-living (low polymer concentration with RNA synthesis dominated by the spontaneous process). These earlier papers focused on RNA replication without considering metabolism. Here, we make a link to metabolism.

**Figure 11:**
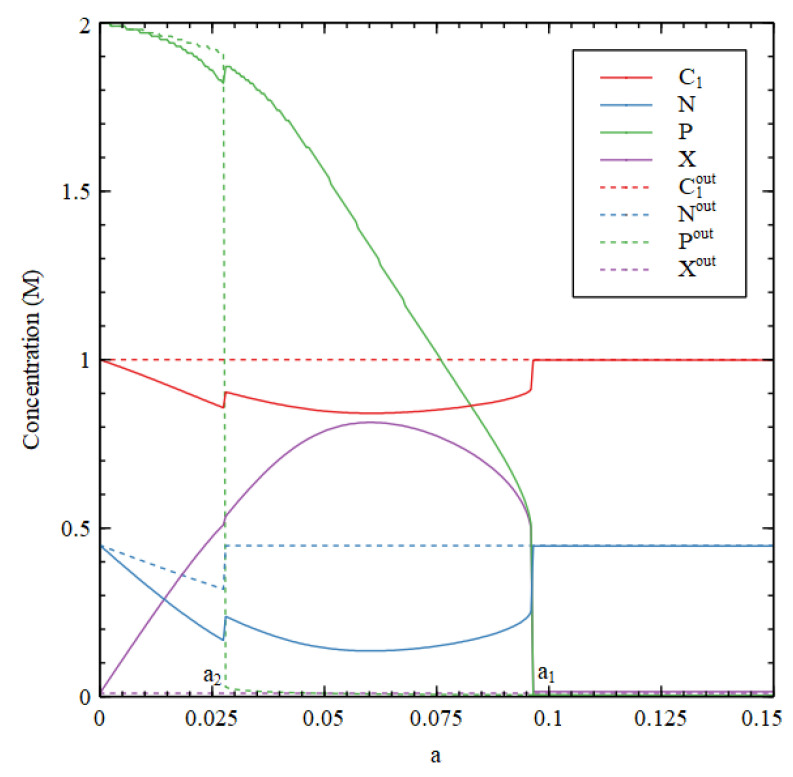
Stationary values of concentrations inside the cell ($C_{n},\;N,\;P,\;X$) and outside the cell ($C_{n}^{out},\;N^{out},\;P^{out},\;X^{out}$) in the RNA synthesis reaction nework as a function of the cleavage rate a.

**Figure 12:**
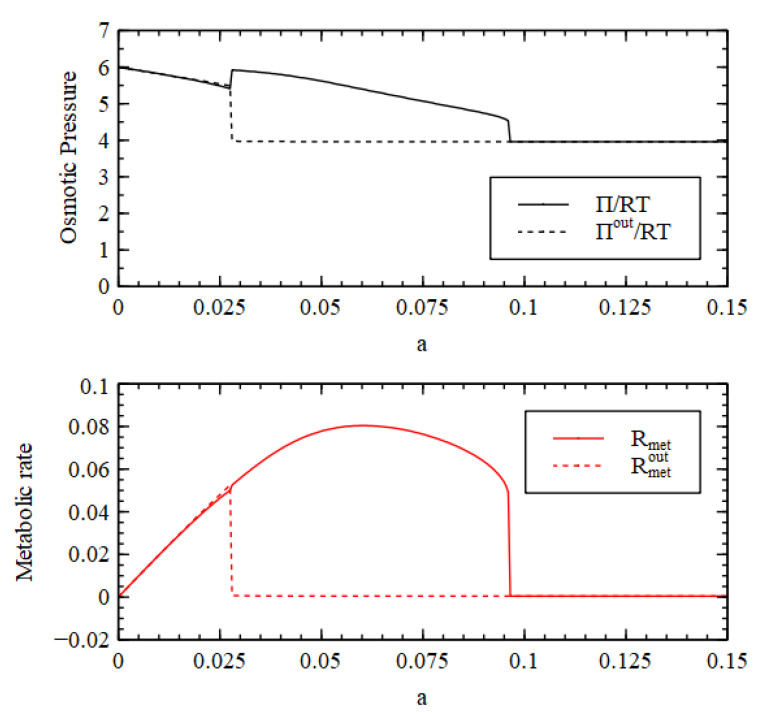
Osmotic pressure and metabolic rate inside $\left(\frac{\mathrm{II}}{RT},\;R_{met}\right)$ and outside the cell $\left(\frac{\mathrm{II}{}^{out}}{RT},\;R_{met}^{out}\right)\;$in the RNA synthesis reaction nework as a function of the cleavage rate a.

Figure 11 and Figure 12 consider a reaction network for RNA synthesis consisting of reactions 1–4 plus polymerization and cleavage reactions. We set $s_{P}=0.001$, $r_{P}=0$, $k_{P}=1$, and $P^{*}=2$, so that the reaction system is bistable and RNA synthesis is predominantly autocatalytic. We consider a fixed-volume compartment. Molecules $A_{1}$ and $A_{2}$ are maintained at constant concentration outside, as before. Molecules $A_{3},\;A_{4}$ and N are all formed outside as well as inside, and are considered as food molecules. The biomolecule is P. The waste molecule is X, which is fixed at a low concentration $X^{out}$= 0.01 M outside.Figure 11 shows the stationary concentrations as a function of cleavage rate. Outside the cell there is an HC and an LC solution, as with the Case 1 small-molecule network, but ‘low’ and ‘high’ refer to the concentration of P in this example because P is the molecule whose presence denotes the living state. There are two transition values in a. For $a>a_{1}$, only the LC concentration is stable. For $a_{2}<a<a_{1}$, both states are stable and the state reached depends on the initial conditions. For $a<a_{2}$, only the HC state is stable. We start the outside in LC initial conditions and the inside in HC initial conditions. Thus, for $a_{2}<a<a_{1}$, the inside goes to the HC state while outside goes to the LC state. For $a>a_{1}$, both inside and outside go to the LC state; for $a<a_{2}$, both inside and outside go to the HC state. Note that $C_{2},\;C_{3},\;C_{4}$ are also present in this example, but for clarity, they are not shown in Figure 11.

The metabolic rate is the polymerization rate in this case $R_{met}=r_{pol}$. We also assume that the single nucleotides are ionized, so they make a contribution to the osmotic pressure which is twice the number of molecules. Under the ideal gas approximation, a polymer contributes only once per chain rather than once per monomer, so the contribution per polymeric nucleotide P is 1+1/l (1 for the ion and 1/l for the polymer). We set the polymer length to be l=50 . The resulting osmotic pressure is

(25) $$\Pi /RT=C_{1}+C_{2}+C_{3}+C_{4}+2\left(N+X\right)+\left(1+1/l\right)P.$$

There are theories giving the osmotic pressure of nucleic acids that are much more acurate than the ideal gas theory [42], but these are more complicated than we require here. The values of these osmotic coefficients do not affect the conclusions of this paper. The main point is that, even though the osmotic coefficient for P is less than that for N, since P is impermeable, the conversion of N to P creates a positive osmotic pressure difference that can drive cell growth. Figure 12 shows that both the osmotic pressure and the metabolic rate are higher inside than out when $a_{2}<a<a_{1}$. Thus, all three criteria for a metabolism are satisfied by the autocatalytic process of RNA polymerization, even though they are not satisfied by the small-molecule reactions that generate the nucleotides. The origin of metabolism occurs only because of the origin of replication in this case.

## 4. Discussion

The three criteria for a metabolism that we proposed here emphasize the maintenance of a difference between the concentration of molecules inside and outside the cell. This difference has been largely ignored previously in both the RAF framework [20,21,22,23], which does not consider concentrations and reaction rates, and in the cyclic process framework [24,25,26,27,28,29,30], because the conditions outside the cell were not considered. The reaction systems studied above have similar properties to networks PM1 and PM2 studied by Piedrafita et al. [30], although the specifications of the reaction networks are different. We now have two examples of bistable networks (PM2 and Case 1) and two examples of monostable networks (PM1 and Case 2). The theory given in this paper makes it clear why the difference between bistable and monostable networks is important. We are assuming that food molecules are present outside and inside the cell, and we are also assuming that the reactions that synthesize biomolecules from food molecules are possible inside the cell. Therefore, it is essential to ask why these same reactions do not occur outside as well. The most natural explanation is that the system is bistable. In that case, there is a stable solution where the biomolecules remain at low concentration outside at the same time as they are at high concentration inside. If the reaction system is monostable, then we need the synthesis of biomolecules to be slow (or prevented) outside the cell or we need the breakdown of biomolecules to be faster outside. While it is possible that these additional differences between inside and outside could exist, we see no compelling reason why they should always exist; hence, they seem like extra conditions that need to be added on *ad hoc*.

One interesting possibility that we have not considered is that physical interactions between molecules could produce some kind of clustering or phase separation leading to the formation of membraneless compartments [43]. We can envisage some kind of polymer with weak attractive interactions that would separate into moderate sized domains without being complete insoluble. If the small-molecule metabolytes are attracted to the phase-separated polymer, then there would naturally be high concentrations and rapid reactions in the high-density regions and low concentrations in the dilute regions. Growth and division would then also require the synthesis of the polymer, so this case is not necessarily simpler than the cases of encapsulation in vesicles that we have already considered. Another similar possibility in the vesicle case is that the small-molecule metabolytes are associated with the surface of the lipid membranes. This would also lead to a increased concentration of metabolytes inside the vesicles (or close to the membranes on both sides). Modifications to lipid molecules can affect their ability to assemble into membranes, so if lipid molecules are involved in metabolic network, then the reactions in a compartment can be strongly coupled to formation, growth and division of the compartment (see [44] for examples).

Bistability has also come up in our previous papers related to the origin of RNA replication [38,39,40,41] in which RNA strands can be synthesized by random polymerization, by non-enzymatic template-directed replication, and by ribozyme-catalyzed replication. When the ribozyme-catalyzed rate is high, there is a bistable system, with an LC and an HC state which we referred to as living and non-living states. We pointed out that the origin of life must occur in a region of parameter space where both states are stable. Obviously life is not possible for rate parameters where only the non-living state is stable. However, the range where only the living state is stable also does not make sense because in that case life would spring up continuously and repeatedly. If both states are stable, then it is possible for life to originate by a chance event that brings together a high concentration of the right molecules in one place. In our previous work [38,39,40] we looked at how long it would take for a stochastic event to cause a compartment in which random RNAs were being synthesized by spontaneous polymerization to jump from the non-living state to the living state, in which ribozyme-catalyzed replication is predominant. Our point was that this jump can occur in a small system with finite volume but not in a well-mixed system with infinite volume. Thus, if the external environment begins in the LC non-living state, then it will remain in that state because concentration fluctuations will be small, whereas in a compartment of small volume, it is possible for a rare stochastic event to initiate a switch to the HC living state. This kind of stochastic transition to the living state could occur with the bistable Case 1 reaction network and with the RNA synthesis network discussed above. A similar effect has also been seen in [26].

There are a lot of similarities between the behaviour of the reaction network in Case 1, and the system for RNA synthesis. At first it appears that there are two transitions in the RNA synthesis model (Figure 11) and only one transition in Case 1 (Figure 3). However, the two transitions in the RNA case occur because we included a small non-zero rate of spontaneous polymerization ($s_{P}=0.001)\;$as well as a high rate of RNA-catalyzed RNA synthesis ($k_{P}=1)$. If the spontaneous rate is set to zero, the transition at $a_{2}$ disappears, and the reaction system is bistable for all $a<a_{1}$. Similarly, in Case 1, we found that there was only one transition at $v_{1}\;$when the direct synthesis rates, $u_{2}$ and $u_{3}$ were zero. However, if we set these rates to a small non-zero rate, then we find that a second transition appears at a decay rate $v_{2}<v_{1}$. Case 1 is then bistable for $v_{2}<v<v_{1}$, while for $v<v_{2}$ only the HC state is stable. This is the same behaviour seen in the RNA model. Another variation of the RNA model not shown here is to allow the linear catalysis term in the polymerization rate $r_{P}P$ to be high, but the quadratic catalysis term $k_{P}P^{2}$ to be zero, which would be true for the simplest descriptions of non-enzymatic template-directed replication in the absence of polymerase ribozymes. In this case the RNA network is autocatalytic but monostable, like Case 2. The reaction network can satisfy the criteria for a metabolism if there is an additional reason why the rate $r_{P}$ is high inside and low outside, or why the cleavage rate a is low inside and high outside.

The analysis of Cases 1,2 and 3 given above reveals the peculiarity that the biomolecules $A_{3},\;A_{4}$ and $A_{5}$ can most easily sustain a metabolism only when their direct synthesis is not possible, as in Case 1. It is only when reactions 2 and 3 do not occur, that the autocatalytic processes involving reactions 4, 5 and 6 become important, and it is these processes that allow the difference between inside and outside the cell to be maintained. This is possibly a reason why small molecules alone cannot easily sustain a metabolism, and why clear experimental examples of small-molecule metabolisms have not been found. It would be necessary to find molecules for which the most obvious pathways of synthesis are blocked for some reason, but where a more complicated autocatalytic synthesis route exists.

The scenario that we have proposed for the RNA synthesis network in Section 3.4 gets us out of this difficulty. For the RNA World to originate, we need a plentiful supply of single nucleotides. The challenge to organic chemists is to find a route for nucleotide synthesis that is simple and direct and prebiotically plausible. This is already quite difficult, although there has been significant progress [45,46,47,48]. However, chemists do not need to solve a problem that would be much more difficult still, namely to synthesize nucleotides via a small-molecule reaction network that is autocatalytic and satisfies the requirements of a metabolism. As we have shown, since template-directed polymerization of RNA (or some similar polymer) is inherently autocatalytic, if the polymerization process gets going inside a protocell, this can sustain the metabolism without requiring prior existence of a small-molecule metabolism. In this scenario, the metabolism would originate only with the establishment of RNA replication, and this could naturally be coupled to growth and division of the cell. In parallel with this suggestion, we note that experimental studies aimed at creating artificial cells usually focus on polymerization of RNA within vesicles [8,9,10,11,12,13], assuming that nucleotides are supplied from the outside. They do not try to develop a small-molecule autocatalytic process for nucleotide synthesis.

The discussion in the previous paragraph is similar to that which arises when considering the origin of homochirality in biomolecules [49]. As nucleic acids and proteins are built from homochiral monomers, and as the synthesis of these monomers and polymers is catalyzed by the homochiral polymers, it is clear that a homochiral polymer system can maintain itself in the homochiral state. It is also possible that a small-molecule system could sustain a homochiral state via asymmetric autocatalysis, but less clear whether this actually occurred in practice prior to the origin of biopolymers. We therefore considered scenarios in which homochirality evolves at the same time as the origin of biopolymer replication, and compared these to scenarios in which the monomers were homochiral prior to the origin of replication [49]. We have since shown that in a model for templating of nucleotides, a symmetry-breaking phase transition can lead to the formation of homochiral RNAs from a racemic mixture of single nucleotides [50]. Thus, homochirality and a self-sustaining metabolism can both arise with the origin of template-directed RNA polymerization, even if neither of these exists at the small-molecule level.

Another general question in models for the origin of life is whether life should begin with a simple autocatalytic process where one kind of molecule makes more of the same thing, or whether it should begin with a collective process in which many kinds of molecules together catalyze the formation of the whole set. If we are discussing small-molecule reaction networks, then there seems to be no reason why the network should not be quite complex. The RAF theories show that autocatalytic sets involving large numbers of molecules are possible. Whether this would arise in practice depends on the details of the real chemical reaction rates, and it is difficult to make concrete predictions from theory. On the other hand, if we are dealing with genetic polymers, then observations of how sequence replication works in modern organisms would suggest that we need something simple first. In modern cells, we observe a simple mechanism for RNA and DNA synthesis that works with all sequences. Similarly, there is a single mechanism for protein synthesis that works with all sequences. We do not see collective mechanisms for replication involving many different mutually-dependent sequences, each of which synthesizes only a small number of other sequences. In our opinion, for the onset of replication, we need a general mechanism that works with all sequences, rather than a complex network of specific reactions that are sequence-specific.

It seems a long way from the simple reaction networks considered in this paper to the complex networks of metabolytes of modern bacteria. The origin of metabolism needs to be linked to the possible chemistry in the prebiotic environment [51,52,53], and we also need to understand how to relate theoretical models of metabolism to the networks in modern bacteria [54,55]. However, it seems odd to use the RAF framework, in which every reaction must be catalyzed, to analyze a reaction system that might have existed prior to the origin of catalysts. In the analysis of bacterial metabolism [54,55], enzyme catalysts were excluded, and when the reactions involved cofactors, the cofactors were treated as the catalysts. For reactions with no cofactors, the authors needed to introduce an imaginary molecule called ‘protein’ to catalyze the reaction, because the existence of a catalyst for every reaction is a requirement of the RAF framework. This seems to be forcing the data to fit the RAF model when it does not naturally do so. We note that cofactors in modern biochemistry participate in reactions by being changed from one form to another (e.g., Coenzyme A gains and loses an acetyl group, or ADP/ATP gains and loses a phosphate), as occurs in the cyclic process framework for reaction dynamics. The cyclic process framework may be a better starting point for analysis of metabolic networks in early organisms. In any case, we would expect that complex networks in which every reaction is controlled by a specific catalyst may have evolved long after the origin of life, and only after the origin of RNA replication and protein synthesis. If some kind of metabolism did evolve early, then it must have been a simple one. The criteria proposed here define what is necessary for a reaction network to support growth and division of a protocell. Satisfying these criteria does not require that every reaction should be catalyzed.

## Figures and Tables

**Table 1:** Reactions and reaction rates in the metabolic system model.

Reaction Number	Reaction	∆G	Reaction Rate	Equilibrium Constant
1	$A_{1}+A_{1}⇄A_{2}$	+0.2*RT*	$r_{1}=u_{1}\left(KC_{1}^{2}-C_{2}\right)$	*K* = exp(−0.2)
2	$A_{1}+A_{2}⇄A_{3}$	+0.2*RT*	$r_{2}=u_{2}\left(KC_{1}C_{2}-C_{3}\right)$	*K* = exp(−0.2)
3	$A_{1}+A_{3}⇄A_{4}$	+0.2*RT*	$r_{3}=u_{3}\left(KC_{1}C_{3}-C_{4}\right)$	*K* = exp(−0.2)
4	$A_{1}+A_{4}⇄A_{5}$	+0.2*RT*	$r_{4}=u_{4}\left(KC_{1}C_{4}-C_{5}\right)$	*K* = exp(−0.2)
5	$A_{2}+A_{4}⇄2A_{3}$	0	$r_{5}=u_{5}\left(C_{2}C_{4}-C_{3}^{2}\right)$	
6	$A_{3}+A_{5}⇄2A_{4}$	0	$r_{6}=u_{6}\left(C_{3}C_{5}-C_{4}^{2}\right)$	
7	$A_{3}⇄3W$	−3.4*RT*	$r_{7}=u_{7}\left(C_{3}-K_{7}W^{3}\right)$	*K*_7_ = exp(−3.4)
8	$A_{4}⇄4W$	−4.6*RT*	$r_{8}=u_{8}\left(C_{4}-K_{8}W^{4}\right)$	*K*_8_ = exp(−4.6)
9	$A_{5}⇄5W$	−5.8*RT*	$r_{9}=u_{9}\left(C_{5}-K_{9}W^{5}\right)$	*K*_9_ = exp(−5.8)

**Table 2:** Values of the reaction rate constants in the different cases analyzed.

Rate Constant	Case 1	Case 2	Case 3
$u_{1}$	1	1	1
$u_{2}$	0	0	1
$u_{3}$	0	1	1
$u_{4}$	1	1	1
$u_{5}$	1	1	0
$u_{6}$	1	0	0
$u_{7}=u_{8}=u_{9}$	*V*	*v*	*v*

**Table 3:** Reactions for lipid synthesis.

Reaction Number	Reaction	∆G	Reaction Rate	Equilibrium Constant
10	$L_{1}+A_{3}⇄L_{2}$	+0.2*RT*	$r_{10}=u_{10}\left(KL_{1}C_{3}-L_{2}\right)$	*K* = exp(−0.2)
11	$L_{2}+A_{1}⇄L+A_{3}$	+0.2*RT*	$r_{11}=u_{11}\left(KL_{2}C_{1}-LC_{3}\right)$	*K* = exp(−0.2)
